# The Type 2 Diabetes Risk Allele of *TMEM154*-rs6813195 Associates with Decreased Beta Cell Function in a Study of 6,486 Danes

**DOI:** 10.1371/journal.pone.0120890

**Published:** 2015-03-23

**Authors:** Marie Neergaard Harder, Emil Vincent Rosenbaum Appel, Niels Grarup, Anette Prior Gjesing, Tarunveer S. Ahluwalia, Torben Jørgensen, Cramer Christensen, Ivan Brandslund, Allan Linneberg, Thorkild I. A. Sørensen, Oluf Pedersen, Torben Hansen

**Affiliations:** 1 The Novo Nordisk Foundation Center for Basic Metabolic Research, Faculty of Health and Medical Sciences, University of Copenhagen, Copenhagen, Denmark; 2 Copenhagen Prospective Studies on Asthma in Childhood, Faculty of Health and Medical Sciences, University of Copenhagen, Copenhagen, Denmark; 3 The Danish Pediatric Asthma Center, Gentofte Hospital, The Capital Region, Copenhagen, Denmark; 4 Research Centre for Prevention and Health, Glostrup University Hospital, Glostrup, Denmark; 5 Department of Public Health, Faculty of Health and Medical Sciences, University of Copenhagen, Copenhagen, Denmark; 6 Medical Department, Lillebaelt Hospital, Vejle Hospital, Vejle, Denmark; 7 Department of Clinical Immunology and Biochemistry, Lillebaelt Hospital, Vejle Hospital, Vejle, Denmark; 8 Institute of Regional Health Research, University of Southern Denmark, Odense, Denmark; 9 Department of Clinical Experimental Research, Glostrup University Hospital, Glostrup, Denmark; 10 Department of Clinical Medicine, Faculty of Health and Medical Sciences, University of Copenhagen, Copenhagen, Denmark; 11 Institute of Preventive Medicine, Bispebjerg and Frederiksberg Hospitals, the Capital Region, Copenhagen, Denmark; 12 Faculty of Health Sciences, University of Southern Denmark, Odense, Denmark; Innsbruck Medical University, AUSTRIA

## Abstract

**Objectives:**

A trans-ethnic meta-analysis of type 2 diabetes genome-wide association studies has identified seven novel susceptibility variants in or near *TMEM154*, *SSR1/RREB1*, *FAF1*, *POU5F1/TCF19*, *LPP*, *ARL15* and *ABCB9/MPHOSPH9*. The aim of our study was to investigate associations between these novel risk variants and type 2 diabetes and pre-diabetic traits in a Danish population-based study with measurements of plasma glucose and serum insulin after an oral glucose tolerance test in order to elaborate on the physiological impact of the variants.

**Methods:**

Case-control analyses were performed in up to 5,777 patients with type 2 diabetes and 7,956 individuals with normal fasting glucose levels. Quantitative trait analyses were performed in up to 5,744 Inter99 participants naïve to glucose-lowering medication. Significant associations between *TMEM154*-rs6813195 and the beta cell measures insulinogenic index and disposition index and between *FAF1*-rs17106184 and 2-hour serum insulin levels were selected for further investigation in additional Danish studies and results were combined in meta-analyses including up to 6,486 Danes.

**Results:**

We confirmed associations with type 2 diabetes for five of the seven SNPs (*TMEM154*-rs6813195, *FAF1*-rs17106184, *POU5F1/TCF19*-rs3130501, *ARL15*-rs702634 and *ABCB9/MPHOSPH9*-rs4275659). The type 2 diabetes risk C-allele of *TMEM154*-rs6813195 associated with decreased disposition index (n=5,181, β=-0.042, *p*=0.012) and insulinogenic index (n=5,181, β=-0.032, *p*=0.043) in Inter99 and these associations remained significant in meta-analyses including four additional Danish studies (disposition index n=6,486, β=-0.042, *p*=0.0044; and insulinogenic index n=6,486, β=-0.037, *p*=0.0094). The type 2 diabetes risk G-allele of *FAF1*-rs17106184 associated with increased levels of 2-hour serum insulin (n=5,547, β=0.055, *p*=0.017) in Inter99 and also when combining effects with three additional Danish studies (n=6,260, β=0.062, *p*=0.0040).

**Conclusion:**

Studies of type 2 diabetes intermediary traits suggest the diabetogenic impact of the C-allele of *TMEM154*-rs6813195 is mediated through reduced beta cell function. The impact of the diabetes risk G-allele of *FAF1*-rs17106184 on increased 2-hour insulin levels is however unexplained.

## Introduction

About 90 genomic loci harboring genetic variation increasing the risk of developing type 2 diabetes (T2D) have been identified mostly by genome-wide association studies (GWAS) [1]. Most large-scale studies have in the past primarily been performed in populations of European decent, and initial genotyping arrays applied in GWAS were designed to mainly capture common genetic variation in Europeans. Recently, there has been an increase in GWAS undertaken in other ethnic groups. This has led to the performance of a multi-ethnic meta-analysis of GWAS data across different populations [2]. By combining GWAS across ancestries, this study enhanced fine-mapping resolution of causal variants because of differences in linkage disequilibrium between populations. Furthermore, this trans-ethnic study had increased power to detect new susceptibility variants as a result of an increased sample size and the authors identified seven novel T2D risk variants. However, the underlying pathophysiology behind the association of these novel variants to T2D is not obvious.

The aim of this study was to investigate the seven novel T2D risk variants discovered in the trans-ethnic meta-analysis for associations with T2D and pre-diabetic quantitative traits in Danish study samples with measurements of glucose and insulin levels after an oral glucose tolerance test (OGTT).

## Materials and Methods

### Study populations

In the case-control analyses, individuals with normal fasting glucose and T2D cases were included from the Danish population-based studies Inter99 [3], Health 2006 study [4], Health 2008 study [5], the outpatient clinic at Steno Diabetes Center, the ADDITION study [6] and Vejle Biobank. Anthropometric data of individuals involved in the case-control analyses are given in S1 Table.

The study of diabetes-related quantitative traits was performed in the Inter99 cohort and significant findings (*p*<0.05) were further analyzed in individuals from the ADIGEN cohort [7] (obese cases and non-obese controls), the Danish Family study [8] and the Health 2008 cohort. Description of the study samples are given in S2 Table and information on anthropometrics and metabolic traits is provided in S3 Table and S4 Table.

### Ethical statement

Written informed consent was obtained from all participants and the protocols were in accordance with the Helsinki Declaration. The studies were approved by the Scientific Ethics Committee of the Capital Region of Denmark (Inter99 KA-98155, Health 2006 KA-20060011, Health 2008 H-KA-20060011, Steno Diabetes Center KA-95117g, KA-94092g, KA-92071 and KA-99081, Danish Family KA-93033 and ADIGEN KF-01–389–97) or the Scientific Ethics Committee of Aarhus County (ADDITION 2000183) or the Scientific Ethics Committee of Southern Denmark (Vejle Biobank S-20080097).

### Metabolic measurements

Participants from Inter99, ADIGEN and Health 2008 without known T2D underwent a 75-g OGTT with blood sampling in the fasting state (after an overnight fast) and at time points 30 min and 120 min. Participants without known T2D from the Danish Family study underwent a 4 hour OGTT with frequent blood sampling (at 10, 20, 30, 40, 50, 60, 75, 90, 105, 120, 140, 160, 180, 210 and 240 min).

Oral glucose-stimulated early insulin response was reported as the insulinogenic index [9] whereas the acute insulin response (AIR) simulating intravenous glucose tolerance test (IVGTT) conditions was estimated by the BIGTT-AIR [10]. Whole-body insulin sensitivity was estimated by the Matsuda insulin sensitivity index (ISI_Matsuda_) [11] and the BIGTT-sensitivity index (SI) [10]. Besides the OGTT-sampled plasma glucose and serum insulin values the BIGTT indexes apply information on sex and body mass index [10]. Beta cell function corrected for whole-body insulin sensitivity level was expressed as the disposition index [12]. Detailed information on metabolic measurements and calculations is provided in S5 Table.

### Genotyping

Genotype information on all seven T2D risk variants (*TMEM154*-rs6813195, *SSR1/RREB1*-rs9505118, *FAF1*-rs17106184, *POU5F1/TCF19*-rs3130501, *LPP*-rs6808574, *ARL15*-rs702634 and *ABCB9/MPHOSPH9*-rs4275659) was available from the Metabochip (Illumina genotyping array) [13], and four of the variants (*TMEM154*-rs6813195, *SSR1/RREB1*-rs9505118, *POU5F1/TCF19*-rs3130501 and *ABCB9/MPHOSPH9*-rs4275659) had also available genotype information from the ExomeBeadChip v1.0 (Illumina genotyping array) [14]. The Inter99, Health 2006 and Steno Diabetes Center cohorts had genotype information from both the Metabochip and the ExomeBeadChip. In these cohorts, the Metabochip genotyping information was used, but if genotypes were missing for any of the four SNPs which were genotyped on both arrays, genotyping information from the ExomeBeadChip was used to supplement where possible. The Health 2008, ADDITION and Vejle Biobank had genotype information from the ExomeBeadChip only and the Danish Family study had genotype information from the Metabochip only. The Metabochip genotype calling and quality control have been described before [15]. ExomeBeadChip genotypes were called using GenCall applying a custom-made cluster file based on 6000 samples with high quality data. From the ExomeBeadChip, quality control of samples and variants was done using PLINK and included exclusion of samples showing relatedness (first- and second-degree relatives), extreme inbreeding coefficient or mismatch between sex status in phenotype and genotype data. Furthermore, we removed individuals with discordant genotype data between one of the four variants available on both genotype arrays (1.5%). Genotyping quality for each selected variant was assessed by the call-rate (>95%) and presence of Hardy-Weinberg equilibrium (P>0.0005).

In ADIGEN, genome-wide genotyping on the Illumina 610k quad chip was carried out at the Centre National deGénotypage (CNG, Evry, France). Quality control and imputation methods have been described previously [16]. The genotypes of *TMEM154*-rs6813195 and *FAF1*-rs17106184 were extracted with a call-rate of 100%.

### Statistical analyses

Analyses in Inter99, Health 2006, Health 2008, ADDITION, Steno Diabetes Center and Vejle Biobank were performed using RStudio software version 0.98.501 (http://www.rstudio.com). In the Danish Family study analyses were performed in SOLAR version 6.6.2 and in the ADIGEN cohort analyses were performed using the R statistical program version 3.0.2 (http://www.r-project.org/).

Case-control analyses: T2D associations were tested by logistic regression using an additive genetic model adjusting for sex and age in up to 5,777 Danish patients with T2D from Inter99 (n = 320), Health 2006 (n = 166), Health 2008 (n = 18), Steno Diabetes Center (n = 1,424), ADDITION (n = 1,870) and Vejle Biobank (n = 1,979) and up to 7,956 individuals with normal fasting glucose from Inter99 (n = 4,590), Health 2006 (n = 2,412), Health 2008 (n = 528) and Vejle Biobank (n = 426).

Quantitative glycemic trait analyses: Associations between the seven novel T2D risk variants and quantitative glycemic traits in up to 5,744 Inter99 individuals without patients known to have T2D were examined by linear regression using additive genetic models adjusting for age (BIGTT indexes) or age and sex (all other traits). Values of serum insulin, insulinogenic index, ISI_Matsuda_, disposition index and BIGTT-AIR were natural log (ln) transformed before analyses. If beta coefficients were back transformed, it was done by e^beta coefficient^ and multiplied by 100 to get effect sizes in percent. The case-control and quantitative trait analyses were beside age and sex also adjusted for BMI in order to obtain estimates independent of adiposity to reduce extra variance that is not attributable to genetic variation or to reveal potentially adiposity modulating effects.

The significant findings (p<0.05) from Inter99 were selected for further analyses in the Health 2008, ADIGEN and Danish Family study cohorts (having OGTT data) where available.

The associations between *TMEM154*-rs6813195 and both insulinogenic index and disposition index were therefore performed in 592 individuals from Health 2008, 165 obese cases and 246 controls from ADIGEN and 302 individuals from the Danish Family study. The association between *FAF1*-rs17106184 and 2-hour serum insulin was also performed in these cohorts except Health 2008, where this variant was not genotyped. The participants from these additional Danish cohorts were all naïve to glucose lowering medication and the analyses using Health 2008 and ADIGEN data were performed by linear regression using additive genetic models adjusting for age, sex and BMI (Health 2008) or age and BMI (ADIGEN). In the 302 individuals from the Danish Family study the analysis was performed using variance components with age, sex and BMI as covariates [17]. Values of insulinogenic index, disposition index and 2-hour serum insulin were ln transformed. The beta coefficients and standard errors derived from the linear regression analyses were then combined in a meta-analysis of up to 6,486 individuals of which 5,181 individuals are from Inter99. The weight of the studies in the meta-analyses was estimated using inverse variance assuming fixed effects. Heterogeneity was measured by Q-statistics.

Bonferroni correction was calculated for 49 independent tests (7 SNPs * 7 traits) corresponding to a new statistical significance threshold of 0.001 (0.05/49).

Statistical power was estimated using 1,000 simulations using the RStudio software version 0.98.501. We used the empirical variance of the observed traits in the Inter99 cohort to simulate phenotypes from a normal distribution, so that variance across genotypes is drawn from the estimated variance. We assumed the risk allele frequency of *TMEM154*-rs6813195 (0.72) and a significance threshold of 0.05.

## Results

### Association with T2D

Three out of the seven T2D risk variants nominally associated with T2D in the Danish population sample of 5,777 cases and 7,956 controls; the C-allele of *TMEM154*-rs6813195 (odds ratio (OR) = 1.09 [95% confidence interval (CI) = 1.02–1.16], *p* = 0.015), the G-allele of *POU5F1/TCF19*-rs3130501 (OR = 1.08 [95% CI = 1.01–1.16], *p* = 0.030) and the C-allele of *MPHOSPH9*-rs4275659 (OR = 1.07 [95% CI = 1.00–1.15], *p* = 0.047) (Table 1). In addition, also the G-allele of *FAF1*-rs17106184 (OR = 1.21 [95% CI = 1.03–1.42], *p* = 0.019) and the A-allele of *ARL15*-rs702634 (OR = 1.12 [95% CI = 1.02–1.25], *p* = 0.024) associated with T2D when also adjusting for BMI (Table 1). All seven variants showed the same direction of effect as originally reported and with comparable odds ratios.

**Table 1:** T2D case-control analyses of up to 5,777 patients from Inter99 (n = 320), Health 2006 (n = 166), Health 2008 (n = 18), Steno Diabetes Center (n = 1,424), ADDITION (n = 1,870) and Vejle Biobank (n = 1,979) and up to 7,956 individuals with normal fasting glucose from Inter99 (n = 4,590), Health 2006 (n = 2,412), Health 2008 (n = 528) and Vejle Biobank (n = 426).

SNP	Function/gene	RA	RAF	n cases vs. n controls	OR (95% CI)	*P*	OR_adjBMI_ (95% CI)	*P*_adjBMI_
rs6813195	Intergenic *TMEM154*	C	0.72	415/2213/3102 vs. 672/3179/4105	1.09 (1.02–1.16)	**0.015**	1.12 (1.03–1.21)	**0.0054**
rs9505118	Intron *SSR1/RREB1*	A	0.59	935/2765/2028 vs. 1318/3920/2716	1.04 (0.98–1.11)	0.21	1.04 (0.97–1.12)	0.25
rs17106184	Intron *FAF1*	G	0.90	10/350/1516 vs. 69/1295/5447	1.09 (0.95–1.26)	0.23	1.21 (1.03–1.42)	**0.019**
rs3130501	Intron *POU5F1/TCF19*	G	0.73	403/2261/3057 vs. 610/3204/4137	1.08 (1.01–1.16)	**0.030**	1.08 (1.00–1.17)	**0.049**
rs6808574	Intergenic *LPP*	C	0.63	224/856/739 vs. 881/3173/2517	1.05 (0.95–1.15)	0.33	1.05 (0.94–1.16)	0.39
rs702634	Intron *ARL15*	A	0.69	172/775/929 vs. 686/2884/3239	1.08 (0.99–1.19)	0.09	1.12 (1.02–1.25)	**0.024**
rs4275659	Intron *ABCB9*/ *MPHOSPH9*	C	0.72	433/2216/3082 vs. 640/3201/4115	1.07 (1.00–1.15)	**0.047**	1.06 (0.99–1.15)	0.11

Number of cases vs. number of controls is shown as 0/1/2 risk alleles. Odds ratios (OR) and *P*-values (*P*) are adjusted for age and sex. OR_adjBMI_ and *P*_adjBMI_ are adjusted for age, sex and BMI. SNP, single nucleotide polymorphism. RA, risk allele. RAF, risk allele frequency. CI, confidence interval.

### Association with metabolic intermediary quantitative traits

**Fig 1:**
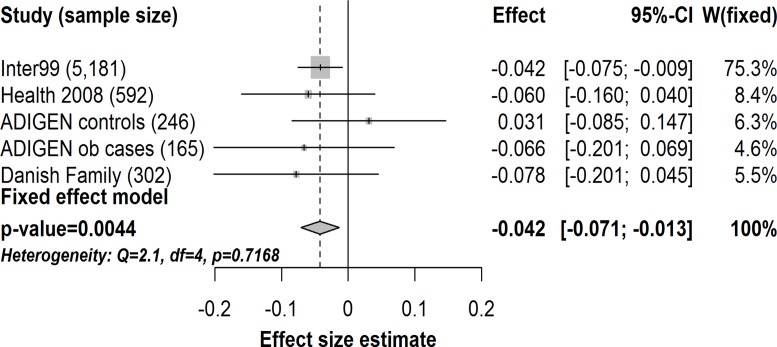
Meta-analysis of the effect of the C-allele of *TMEM154*-rs6813195 on disposition index in 6,486 individuals from the Inter99 study (n = 5,181), Health 2008 study (n = 592), ADIGEN controls (n = 246), ADIGEN obese cases (n = 165) and Danish Family study (n = 302). Gray diamond represents combined change per risk allele and the 95% confidence interval. Gray squares represent effects size estimates (beta coefficients) in single studies sized according to their weight in the meta-analyses. The horizontal lines through the gray squares represent the 95% confidence interval. ob, obese. *p*, *P*-value. CI, confidence interval. W(fixed), study weight in the fixed effect meta-analysis.

Carriers of the T2D risk C-allele of *TMEM154*-rs6813195 had a lower disposition index in Inter99 both in the analysis with (n = 5,181, β = -0.042, *p* = 0.012) and without adjustments for BMI (n = 5,182, β = -0.043, *p* = 0.014) (Table 2). The association remained significant with an effect size estimate similar to what we observed in Inter99 when combining data from Inter99, Health 2008, ADIGEN and the Danish Family study in a meta-analysis (n = 6,486, β = -0.042, *p* = 0.0044) (Fig. 1). In other words, carriers of the T2D risk C-allele of *TMEM154*-rs6813195 had on average a 4.1% lower value of the disposition index per C-allele.

**Table 2:** Associations between the seven T2D risk variants and quantitative traits in up to 5,744 Danish individuals naive to glucose-lowering medication.

*TMEM154* rs6813195	TT	TC	CC	Effect	SE	*P*	*P*_adjBMI_
n (% men/women)	469 (46/54)	2,281 (51/49)	2,994 (49/51)				
Age (years)	45 (40–50)	45 (40–50)	45 (40–50)				
BMI (kg/m^2^)	26 (23–28)	26 (23–28)	26 (23–29)	0.001	0.003	0.74	-
30-min insulin (pmol/l)	251 (177–357)	252 (176–358)	243 (173–352)	-0.010	0.012	0.39	0.32
2-hour insulin (pmol/l)	165 (99–264)	151 (92–252)	159 (99–259)	0.015	0.017	0.36	0.31
Insulinogenic index	77.1 (46.3–126.0)	74.0 (46.1–119.9)	69.0 (43.9–117.0)	-0.031	0.016	0.052	**0.043**
ISI_Matsuda_	7.6 (5.2–11.0)	7.9 (5.2–11.3)	7.7 (5.1–11.3)	-0.009	0.013	0.49	0.64
Disposition index	546 (319–986)	547 (333–948)	518 (313–877)	-0.043	0.017	**0.014**	**0.012**
BIGTT-*S*_I_	9.2 ± 3.9	9.3 ± 4.1	9.2 ± 4.1	-0.014	0.088	0.87	-
BIGTT-AIR	1,656 (1,288–2,163)	1,642 (1,305–2,085)	1,613 (1,277–2,069)	-0.010	0.009	0.27	-
***SSR1/RREB1* rs9505118**	**GG**	**GA**	**AA**	**Effect**	**SE**	***P***	
n (% men/women)	957 (50/50)	2,804 (50/50)	1,982 (49/51)				
Age (years)	45 (40–50)	45 (40–50)	45 (40–50)				
BMI (kg/m^2^)	26 (23–28)	26 (23–29)	26 (23–28)	0.000	0.003	0.90	-
30-min insulin (pmol/l)	254 (176–360)	246 (175–353)	244 (174–354)	-0.002	0.011	0.82	0.79
2-hour insulin (pmol/l)	155 (96–265)	158 (97–253)	155 (98–256)	0.004	0.015	0.79	0.83
Insulinogenic index	73.3 (45.5–120.2)	71.1 (44.7–115.7)	71.3 (44.5–122.6)	0.003	0.015	0.85	0.87
ISI_Matsuda_	7.8 (5.2–10.9)	7.7 (5.2–11.3)	7.9 (5.2–11.4)	0.008	0.012	0.52	0.35
Disposition index	532 (321–898)	525 (316–902)	544 (326–940)	0.007	0.016	0.68	0.69
BIGTT-*S*_I_	9.2 ± 4.0	9.2 ± 4.1	9.3 ± 4.0	0.019	0.081	0.82	-
BIGTT-AIR	1,643 (1,295–2,042)	1,623 (1,284–2,078)	1,630 (1,279–2,097)	0.005	0.009	0.53	-
***FAF1* rs17106184**	**AA**	**AG**	**GG**	**Effect**	**SE**	***P***	
n (% men/women)	53 (60/40)	1,068 (47/53)	4,506 (50/50)				
Age (years)	45 (40–55)	45 (40–50)	45 (40–50)				
BMI (kg/m^2^)	26 (24–28)	26 (23–29)	26 (23–28)	-0.006	0.005	0.24	-
30-min insulin (pmol/l)	247 (185–300)	253 (175–364)	245 (175–354)	-0.008	0.018	0.65	0.88
2-hour insulin (pmol/l)	132 (92–287)	150 (93–248)	159 (98–257)	0.046	0.025	0.07	**0.017**
Insulinogenic index	65.4 (43.1–108.2)	72.6 (45.6–123.0)	71.4 (44.7–118.7)	-0.008	0.024	0.73	0.85
ISI_Matsuda_	7.7 (5.2–9.8)	7.8 (5.1–11.3)	7.8 (5.2–11.3)	0.006	0.019	0.73	0.64
Disposition index	508 (279–882)	528 (333–908)	535 (318–909)	-0.003	0.026	0.90	0.69
BIGTT-*S*_I_	8.9 ± 3.4	9.3 ± 4.0	9.2 ± 4.1	-0.020	0.133	0.88	-
BIGTT-AIR	1,558 (1,448–2,028)	1,637 (1,295–2,077)	1,627 (1,283–2,080)	0.006	0.014	0.68	-
***POU5F1/TCF19* rs3130501**	**AA**	**AG**	**GG**	**Effect**	**SE**	***P***	
n (% men/women)	434 (52/48)	2,263 (49/51)	3,045 (50/50)				
Age (years)	45 (40–55)	45 (40–50)	45 (40–50)				
BMI (kg/m^2^)	26 (24–29)	25 (23–28)	26 (23–29)	-0.002	0.003	0.51	-
30-min insulin (pmol/l)	262 (185–357)	241 (175–342)	248 (173–360)	-0.011	0.012	0.37	0.56
2-hour insulin (pmol/l)	159 (97–264)	154 (95–251)	158 (98–260)	0.009	0.017	0.58	0.43
Insulinogenic index	70.0 (45.4–116.7)	71.4 (44.6–117.5)	71.8 (44.7–120.0)	-0.001	0.016	0.93	1.00
ISI_Matsuda_	7.4 (5.2–10.5)	7.8 (5.2–73.0)	7.8 (5.2–11.3)	0.011	0.013	0.38	0.48
Disposition index	518 (315–800)	536 (321–927)	532 (322–918)	0.005	0.018	0.77	0.91
BIGTT-*S*_I_	8.8 ± 3.8	9.3 ± 4.1	9.2 ± 4.0	0.016	0.089	0.86	-
BIGTT-AIR	1,635 (1,295–2,166)	1,639 (1,290–2,079)	1,622 (1,281–2,074)	-0.007	0.009	0.47	-
***LPP* rs6808574**	**TT**	**TC**	**CC**	**Effect**	**SE**	***P***	
n (% men/women)	720 (49/51)	2,634 (51/49)	2,098 (49/51)				
Age (years)	45 (40–50)	45 (40–50)	45 (40–50)				
BMI (kg/m^2^)	25 (23–28)	26 (23–29)	26 (23–28)	-0.001	0.003	0.74	-
30-min insulin (pmol/l)	250 (171–363)	247 (176–356)	243 (176–353)	-0.001	0.011	0.96	0.96
2-hour insulin (pmol/l)	159 (95–263)	157 (98–262)	156 (97–248)	-0.007	0.016	0.67	0.72
Insulinogenic index	73.0 (45.3–121.9)	70.9 (44.1–120.4)	71.6 (45.8–116.1)	-0.009	0.016	0.55	0.60
ISI_Matsuda_	7.5 (5.1–11.2)	7.8 (5.1–11.2)	7.8 (5.3–11.4)	0.016	0.012	0.19	0.23
Disposition index	510 (320–902)	531 (314–921)	539 (327–900)	0.007	0.017	0.69	0.77
BIGTT-*S*_I_	9.2 ± 4.1	9.1 ± 4.1	9.3 ± 4.0	0.074	0.086	0.39	-
BIGTT-AIR	1,651 (1,301–2,131)	1,627 (1,270–2,073)	1,621 (1,296–2,072)	-0.010	0.009	0.29	-
***ARL15* rs702634**	**GG**	**GA**	**AA**	**Effect**	**SE**	***P***	
n (% men/women)	523 (50/50)	2,419 (49/51)	2,684 (51/49)				
Age (years)	45 (40–50)	45 (40–50)	45 (40–50)				
BMI (kg/m^2^)	26 (23–29)	26 (23–29)	26 (23–28)	-0.005	0.003	0.13	-
30-min insulin (pmol/l)	257 (174–349)	244 (174–354)	247 (176–359)	0.003	0.012	0.81	0.40
2-hour insulin (pmol/l)	152 (96–251)	157 (97–256)	156 (96–256)	0.014	0.017	0.41	0.16
Insulinogenic index	72.3 (45.5–119.1)	71.2 (44.7–120.5)	71.4 (44.7–117.6)	-0.014	0.016	0.39	0.51
ISI_Matsuda_	8.0 (5.4–11.3)	7.8 (5.2–11.1)	7.7 (5.1–11.4)	-0.005	0.012	0.68	0.19
Disposition index	573 (337–957)	542 (322–902)	516 (317–908)	-0.019	0.017	0.26	0.12
BIGTT-*S*_I_	9.3 ± 4.1	9.1 ± 4.0	9.2 ± 4.1	-0.030	0.087	0.73	-
BIGTT-AIR	1,661 (1,311–2,065)	1,623 (1,293–2,085)	1,622 (1,272–2,075)	-0.007	0.009	0.47	-
***MPHOSPH9* rs4275659**	**TT**	**TC**	**CC**	**Effect**	**SE**	***P***	
n (% men/women)	462 (47/53)	2,265 (50/50)	3,017 (50/50)				
Age (years)	45 (40–50)	45 (40–50)	45 (40–50)				
BMI (kg/m^2^)	25 (23–28)	26 (23–29)	26 (23–29)	0.002	0.003	0.63	-
30-min insulin (pmol/l)	244 (178–359)	243 (176–352)	249 (173–358)	-0.007	0.012	0.55	0.32
2-hour insulin (pmol/l)	154 (94–260)	156 (97–254)	157 (97–256)	0.014	0.017	0.41	0.47
Insulinogenic index	70.3 (44.3–119.8)	71.0 (45.3–117.5)	72.2 (44.7–119.3)	-0.005	0.016	0.77	0.65
ISI_Matsuda_	8.0 (5.1–11.5)	7.9 (5.2–11.3)	7.7 (5.2–11.2)	-0.014	0.013	0.25	0.32
Disposition index	570 (335–961)	532 (316–908)	528 (323–907)	-0.018	0.017	0.31	0.44
BIGTT-*S*_I_	9.4 ± 4.1	9.2 ± 4.1	9.1 ± 4.0	-0.127	0.088	0.15	-
BIGTT-AIR	1,615 (1,265–2,080)	1,627 (1,277–2,077)	1,632 (1,300–2,086)	0.006	0.009	0.52	-

Raw data are mean±SD or median (interquartile range) and are stratified according to genotype. Values of serum insulin and derived indexes of insulinogenic index, ISI_Matsuda_, disposition index and BIGTT-AIR were natural logarithmical (ln) transformed before analysis. Effects represent beta coefficients and are shown for the T2D risk allele. *P*-values (*P*) are adjusted for age (BIGTT-AIR and BIGTT-SI) or sex and age (all other traits). *P*_adjBMI_ are *P*-values adjusted for age, sex and BMI. All analyses assume an additive genetic model. SE, standard error.

**Fig 2:**
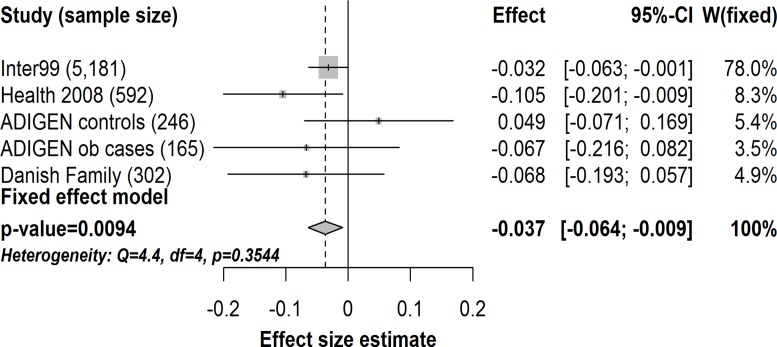
Meta-analysis of the effect of the C-allele of *TMEM154*-rs6813195 on insulinogenic index in 6,486 individuals from the Inter99 study (n = 5,181), Health 2008 study (n = 592), ADIGEN controls (n = 246), ADIGEN obese cases (n = 165) and Danish Family study (n = 302). Gray diamond represents combined change per risk allele and the 95% confidence interval. Gray squares represent effects size estimates (beta coefficients) in single studies sized according to their weight in the meta-analyses. The horizontal lines through the gray squares represent the 95% confidence interval. ob, obese. *p*, *P*-value. CI, confidence interval. W(fixed), study weight in the fixed effect meta-analysis.

We also found a borderline significant association between the C-allele of *TMEM154*-rs6813195 and a lower insulinogenic index again both in the analysis with (n = 5,181, β = -0.032, *p* = 0.043) and without adjustments for BMI (n = 5,182, β = -0.031, *p* = 0.052) in Inter99. In the meta-analysis combining data from Inter99, Health 2008, ADIGEN and the Danish Family study the association with insulinogenic index also remained significant with a similar effect size estimate (n = 6,486, β = -0.037, *p* = 0.0094) (Fig. 2). This corresponds to an average lower value of insulinogenic index of 3.6% per C-allele in *TMEM154*-rs6813195 C-allele carriers.

**Fig 3:**
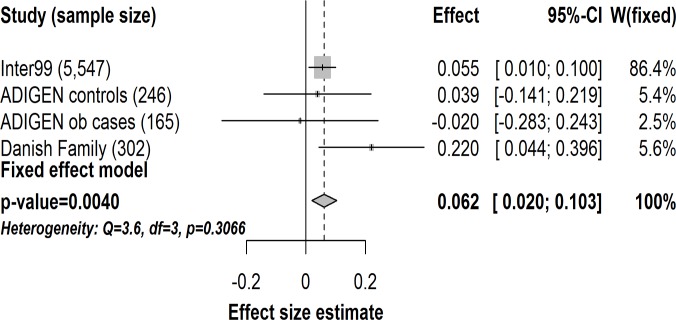
Meta-analysis of the effect of the G-allele of *FAF1*-rs17106184 on 2-hour serum insulin in 6,260 individuals from the Inter99 study (n = 5,547), ADIGEN controls (n = 246), ADIGEN obese cases (n = 165) and Danish Family study (n = 302). Gray diamond represents combined change per risk allele and the 95% confidence interval. Gray squares represent effects size estimates (beta coefficients) in single studies sized according to their weight in the meta-analyses. The horizontal lines through the gray squares represent the 95% confidence interval. ob, obese. *p*, *P*-value. CI, confidence interval. W(fixed), study weight in the fixed effect meta-analysis.

Furthermore, carriers of the T2D risk G-allele of *FAF1*-rs17106184 had an increased level of serum insulin 2 hours after an oral glucose challenge when the analysis was adjusted for BMI (n = 5,547, β = 0.055, *p* = 0.017). When this association was tested in ADIGEN and the Danish Family study and results were combined in a meta-analysis, it also remained significant with a similar effect size estimate (n = 6,260, β = 0.062, *p* = 0.0040) (Fig. 3). This means that carries of the *FAF1*-rs17106184 G-allele on average had a 6.4% higher level of 2-hour insulin per G-allele. We did not find any associations between the remaining five T2D risk variants and the diabetes-related traits (30-min serum insulin, 2-hour serum insulin, insulinogenic index, ISI_Matsuda_, disposition index, BIGTT-*S*_I_ and BIGTT-AIR).

## Discussion

In most large populations used for genome-wide association studies, only measures of fasting plasma glucose and fasting serum insulin levels are available, making it difficult to link susceptibility SNPs with a detailed pre-diabetic quantitative phenotype. For some of the SNPs, follow-up studies in smaller populations with more elaborated measures of glycemic traits have proposed an underlying physiological phenotype [18]. In this study we investigated the effect of seven novel T2D risk variants on surrogate measures of beta cell function and insulin sensitivity in order to elaborate on their cause of disease association. We found associations between a metabolic intermediate trait and two out of the seven T2D risk variants (*TMEM154*-rs6813195 and *FAF1*-rs17106184).

Carriers of *TMEM154*-rs6813195 C-allele had on average a decreased disposition index in the Inter99 cohort. The relationship between insulin sensitivity and insulin secretion is thought to be approximately hyperbolic so that the product of the two variables is constant for individuals with the same degree of glucose tolerance [12]. This constant is known as the disposition index and is thus an indication of the ability of the beta cells to respond appropriately to the level of insulin resistance. A low value of the disposition index can be caused by a continual exposure to triggers of insulin resistance (e.g. high fat diet or lack of exercise) without beta cell compensation and results in the progression to pre-diabetes or even to overt T2D. When we further examined the association between *TMEM154*-rs6813195 and disposition index in the additional smaller cohorts, we found the same direction of effect. The variant also showed a borderline significant association with decreased insulinogenic index in Inter99 and with the same direction of effect in the meta-analysis. The insulinogenic index is a ratio relating enhancement of circulating insulin to magnitude of corresponding glycemic stimulus and is thus a measure of early insulin response [9]. This observation supports the suggestive negative role of *TMEM154*-rs6813195 on beta cell function. The *TMEM154*-rs6813195 did not associate with any fasting glycemic traits in the MAGIC consortium [2].

*TMEM154* codes for a transmembrane protein, showing ubiquitous mRNA expression highest in B lymphocytes (Human geneAtlas [19]) but immunohistochemical staining of human gastrointestinal tract shows strong cytoplasmic and membranous positivity of TMEM154 in glandular cells of the digestive tract including the duodenum (The Human Protein Atlas [20]). If these glandular cells are of enteroendocrine character, it can be suggested that the variant near *TMEM154* therefore might have an effect on intestinal secretion of hormones affecting pancreatic beta cells. If this is the case and the variant has an effect on secretion of hormones such as incretin hormones, then the effect would be observed by an OGTT and not by an IVGTT. In our data this variant does not associate with BIGTT-AIR, which is a measure of insulin release simulating IVGTT conditions, fitting well to the suggestive interpretation of data. However, the fact that we do not observe an association with BIGTT-AIR can also be due to lack of statistical power.

Carriers of *FAF1*-rs17106184 G-allele had on average an increased level of insulin 2 hours after a glucose challenge. A neighbouring gene to *FAF1* is *ELAVL4* (also called *HuD*), which is expressed in the pancreas. The product of *HuD* binds to insulin mRNA 5’-UTR and represses translation of insulin [21]. If *FAF1*-rs17106184 has a negative effect on expression of *HuD*, this could result in a decreased repression of insulin translation and therefore in more insulin being produced and later also released explaining the observed increased insulin levels. It is however unexpected that the T2D risk allele associates with increased insulin secretion, when it does not seem to associate with increased insulin resistance either in our or MAGIC consortium data [2].

When interpreting our results it should be noticed that if P-values were corrected for testing seven SNPs and seven traits, none of the observed associations would remain significant. This is however a very stringent correction, since the seven traits are highly correlated. This study is a cross-sectional study and the associations found here are related to a certain time-point and have a poor clinical value like the rest of the T2D-associated variants identified by GWAS due to their small effect sizes. It is not known if our observed associations could change over time being more clinical relevant, but so far longitudinal studies of T2D risk prediction including genetic variants have reported similar results to those from cross-sectional studies [22].

Still larger sample sizes are used in meta-analyses of T2D GWAS in order to discover novel susceptibility loci. In 2012 Morris *et al* performed a meta-analysis of Metabochip data involving 34,840 T2D cases and 114,981 controls [23]. In the latest trans-ethnic T2D meta-analysis this number has increased to 47,979 T2D cases and 139,611 controls [2]. As a consequence the larger sample size makes it possible to identify T2D risk variants with smaller effect sizes (odds ratios <1.10). Similarly, the effects of the GWAS-identified variants on quantitative metabolic traits are likely smaller compared to the risk variants discovered by the first wave of GWAS. In this study using Inter99 data we generally observed small effect sizes with narrow 95% confidence intervals (often in the range of +/- 0.03 (log-transformed units) around the beta coefficient except for BIG-SI), indicating that possible true effect sizes likely reflect very small differences between carriers and non-carriers. The more well-characterized study populations are often of smaller size and will eventually run out of statistical power to unravel the potential mechanism of novel variants with very small effects. In the Inter99 cohort we have 80% statistical power to find per allele effect sizes down to 0.055 for disposition index and down to 0.035 for ISI_Matsuda_ for a common variant with a risk allele frequency of 72%. If the novel variants have a very small true effect on the trait tested here, the few findings of associations in the present study can be explained by low statistical power. Another explanation of the sparse findings could be that the association signals in these loci inflict risk of T2D by yet unknown pathophysiological pathways not measured by the phenotypes we analyzed. It is also possible that the limited statistical model of this study is the major bottleneck, and that only certain gene-environment interactions models can reveal the effects of these variants.

In conclusion, if replicated in independent study materials our explorative examinations of intermediary phenotypes of seven recently discovered T2D genomic risk loci suggest that carriers of the T2D risk C-allele of *TMEM154*-rs6813195 have a decreased ability to secrete sufficient amount of insulin after an oral glucose load. Furthermore, much larger study materials, additional and refined phenotyping or novel statistical modeling seem to be needed to detect the pathophysiological implications of newly identified T2D gene variants with modest effect sizes.

## Supporting Information

S1 Table Anthropometric data of individuals used in the case-control analyses. The individuals are from six different cohorts; Inter99, Health 2006, Health 2008, Steno Diabetes Center (SDC), ADDITION and Vejle Biobank and are stratified by case (T2D) and control (normal fasting glucose) status. Data are median (interquartile range).
(DOCX)

S2 Table Study descriptions of the Danish cohorts used in the study. (DOCX)

S3 Table Anthropometrics and metabolic traits of Inter99 participants naïve to glucose-lowering medication used for pre-diabetic quantitative trait analyses. Raw data are shown for individuals with available DNA and are median (interquartile range) or mean±SD. NGT, normal glucose tolerance. IFG, impaired fasting glucose. IGT, impaired glucose tolerance. scT2D, screen detected type 2 diabetes.
(DOCX)

S4 Table Anthropometrics and metabolic traits of Health 2008, Danish Family and ADIGEN participants naïve to glucose-lowering medication used for pre-diabetic quantitative trait analyses. Raw data are median (interquartile range) or mean±SD. NGT, normal glucose tolerance. IFG, impaired fasting glucose. IGT, impaired glucose tolerance. scT2D, screen detected type 2 diabetes.
(DOCX)

S5 Table Measurements of serum insulin and calculation of glycemic indexes. (DOCX)
