# Supplementary material for: The Type 2 Diabetes Risk Allele of TMEM154-rs6813195 Associates with Decreased Beta Cell Function in a Study of 6,486 Danes
Source: PLoS One. 2015 Mar 23;10(3):e0120890. doi: 10.1371/journal.pone.0120890 (PMC4370672; doi:10.1371/journal.pone.0120890)
Supplement: S1 Table — The individuals are from six different cohorts; Inter99, Health 2006, Health 2008, Steno Diabetes Center (SDC), ADDITION and Vejle Biobank and are stratified by case (T2D) and control (normal fasting glucose) status. Data are median (interquartile range). (DOCX) [file pone.0120890.s001.docx]

**S1 Table.** Anthropometric data of individuals used in the case-control analyses.

|  |  | **N (% male/female)** | **Age in years** | **BMI kg/m^2^** |
| --- | --- | --- | --- | --- |
| **Inter99** | Cases | 320 (62/38) | 50 (45-55) | 29.4 (26.6-33.7) |
|  | Controls | 4,590 (45 /55) | 45 (40-50) | 25.0 (22.8-27.7) |
| **Health 2006** | Cases | 166 (60/40) | 60 (52-66) | 28.0 (25.7-32.7) |
|  | Controls | 2,412 (42/58) | 49 (40-60) | 24.9 (22.4-27.9) |
| **Health 2008** | Cases | 18 (78/22) | 57 (53-59) | 29.5 (25.0-31.0) |
|  | Controls | 528 (41/59) | 45 (39-52) | 24.4 (22.3-27.1) |
| **SDC** | Cases | 1,424 (63/37) | 64 (56-71) | 29.4 (26.5-33.2) |
|  | Controls | 0 | - | - |
| **ADDITION** | Cases | 1,870 (56/44) | 61 (56-66) | 30.5 (27.5-34.0) |
|  | Controls | 0 | - | - |
| **Vejle Biobank** | Cases | 1,979 (62/38) | 64 (58-70) | 29.8 (26.7-33.2) |
|  | Controls | 426 (34/66) | 61 (49-68) | 22.3 (21.1-23.8) |

The individuals are from six different cohorts; Inter99, Health 2006, Health 2008, Steno Diabetes Center (SDC), ADDITION and Vejle Biobank and are stratified by case (T2D) and control (normal fasting glucose) status. Data are median (interquartile range).
