# Supplementary material for: The Type 2 Diabetes Risk Allele of TMEM154-rs6813195 Associates with Decreased Beta Cell Function in a Study of 6,486 Danes
Source: PLoS One. 2015 Mar 23;10(3):e0120890. doi: 10.1371/journal.pone.0120890 (PMC4370672; doi:10.1371/journal.pone.0120890)
Supplement: S2 Table — (DOCX) [file pone.0120890.s002.docx]

**S2 Table.** Study descriptions of the Danish cohorts used in the study

| **Inter99** | The Inter99 study is a Danish population-based study, which was originally designed as a non-pharmacological intervention study for the prevention of ischemic heart disease conducted at the Research Centre for Prevention and Health at Glostrup Hospital, Capital Region of Denmark (ClinicalTrials.gov ID-no: NCT00289237, [www.inter99.dk](http://www.inter99.dk)). A random sample of individuals aged 30-60 years and living in the south western part of Copenhagen was drawn from the general population by the Danish Central Personal Register. A total of 6,784 individuals participated in the study and were Danes by self-report. |
| --- | --- |
| **Health 2006** | Health 2006 is a population-based study conducted at the Research Centre for Prevention and Health at Glostrup Hospital, Capital Region of Denmark. Participants were drawn as a random sample from the general population obtained from the Danish Central Personal Register aged 18-69 years living in 11 municipalities in the south-western part of Copenhagen. 3,471 individuals participated in the general health examination which took place between June 2006 and June 2008. All were Danish citizen and born in Denmark. |
| **Health 2008** | The Health 2008 is an extension of the Health 2006 study and was conducted at the Research Centre for Prevention and Health at Glostrup Hospital, Capital Region of Denmark. A random sample of the general population aged 30 to 60 years living in 10 municipalities in the western part of Capital Region of Denmark was drawn and invited to participate. A total of 795 were examined which took place from September 2008 to December 2009. |
| **SDC** | This biobank consist of patients with type 2 diabetes recruited from the outpatient clinic at Steno Diabetes Center (SDC). |
| **ADDITION** | The ADDITION (Anglo-Danish-Dutch study of Intensive Treatment In PeOple with screen detected diabetes in primary care) is a multi-factorial intervention study which focuses on morbidity and mortality among people with type 2 diabetes. 3000 people aged 40-69 years in three different European countries participate in the study. |
| **Vejle Biobank** | This biobank contains 3000 individuals with type 2 diabetes, 600 individuals with type 1 diabetes and 5000 controls, who are matched on age and sex. Patients were recruited from the central database at Vejle Hospital Laboratory Center to investigate if the development of late diabetic complications and lack of treatment effect have a genetic cause. |
| **ADIGEN** | The ADIGEN (Adiposity Genetics) participants are a subset of a longitudinal case-cohort (obese, non-obese) dataset. This dataset comprise all obese participants (BMI ≥31.0 kg/m2) and 1% randomly chosen non-obese controls selected after examination of 362,200 Caucasian men at the mean age of 20 years at the draft boards in Copenhagen and its surrounding areas during 1943–1977. Obesity was defined as 35% overweight relative to a local standard in use at the time (mid 1970’s). Controls were selected from the same cohort excluding obese individuals already included in the obese group. All obese and half of the controls were invited to re-examinations as part of the Copenhagen City Heart Study surveys in 1982–84 and 1992–93. The AIDGEN study included 568 participants (248 obese and 320 controls) with an age range between 40-65 years. The criteria for invitation to the ADIGEN follow-up surveys and participation have been described previously [[7](#_ENREF_7)]. In total, 194 obese and 290 controls underwent an OGTT. |
| **Danish Family Study** | The Danish Family study consists of 533 individuals from 95 families with one parent suffering from verified type 2 diabetes and one parent without known diabetes. Patients were identified through the outpatient clinic at Steno Diabetes Center (Gentofte, Denmark) or through an ongoing family study at the University of Copenhagen (Copenhagen, Denmark). All probands had diabetes onset after 40 years of age and no known family history of type 1 diabetes. All non-diabetic family members (spouses, offspring and other relatives) were asked to participate in the study. In total, 329 individuals underwent a 4 hour OGTT. |
