# Supplementary material for: The Type 2 Diabetes Risk Allele of TMEM154-rs6813195 Associates with Decreased Beta Cell Function in a Study of 6,486 Danes
Source: PLoS One. 2015 Mar 23;10(3):e0120890. doi: 10.1371/journal.pone.0120890 (PMC4370672; doi:10.1371/journal.pone.0120890)
Supplement: S3 Table — Raw data are shown for individuals with available DNA and are median (interquartile range) or mean±SD. NGT, normal glucose tolerance. IFG, impaired fasting glucose. IGT, impaired glucose tolerance. scT2D, screen detected type 2 diabetes. (DOCX) [file pone.0120890.s003.docx]

**S3 Table**. Anthropometrics and metabolic traits of Inter99 participants naïve to glucose-lowering medication used for pre-diabetic quantitative trait analyses.

| **Characteristics** | **Inter99** |
| --- | --- |
| N (% male/female) | 5,744 (50/50) |
| N NGT/IFG/IGT/scT2D | 4,352/479/670/243 |
| % NGT/IFG/IGT/scT2D | 76/8.5/11.5/4 |
| Age (years) | 45 (40-50) |
| BMI (kg/m^2^) | 25.6 (23.2-28.5) |
| 30-min insulin (pmol/l) | 246 (175-355) |
| 2 hour insulin (pmol/l) | 156 (96-256) |
| Insulinogenic index | 71.5 (44.7-119.0) |
| ISI_Matsuda_ | 7.8 (5.2-11.3) |
| Disposition index | 533 (320-910) |
| BIGTT-SI | 9.2 ± 4.1 |
| BIGTT-AIR | 1,629 (1,285-2,082) |

Raw data are shown for individuals with available DNA and are median (interquartile range) or mean±SD. NGT, normal glucose tolerance. IFG, impaired fasting glucose. IGT, impaired glucose tolerance. scT2D, screen detected type 2 diabetes.
