# Supplementary material for: The Type 2 Diabetes Risk Allele of TMEM154-rs6813195 Associates with Decreased Beta Cell Function in a Study of 6,486 Danes
Source: PLoS One. 2015 Mar 23;10(3):e0120890. doi: 10.1371/journal.pone.0120890 (PMC4370672; doi:10.1371/journal.pone.0120890)
Supplement: S4 Table — Raw data are median (interquartile range) or mean±SD. NGT, normal glucose tolerance. IFG, impaired fasting glucose. IGT, impaired glucose tolerance. scT2D, screen detected type 2 diabetes. (DOCX) [file pone.0120890.s004.docx]

**S4 Table**. Anthropometrics and metabolic traits of Health 2008, Danish Family and ADIGEN participants naïve to glucose-lowering medication used for pre-diabetic quantitative trait analyses.

| **Characteristics** | **Health 2008** | **Danish Family** | **ADIGEN controls** | **ADIGEN cases** |
| --- | --- | --- | --- | --- |
| N (% male/female) | 592 (47/53) | 302 (42/58) | 246 (100/0) | 165 (100/0) |
| N NGT/IFG/IGT/scT2D | 488/54/36/14 | 256/8/38 | 167/26/53/0 | 92/15/58/0 |
| % NGT/IFG/IGT/scT2D | 82.4/9.1/6.1/2.4 | 85/2.5/12.5/0 | 68/10.5/21.5/0 | 56/9/35/0 |
| Age (years) | 47 (41-53) | 40 (34-51) | 49 (45-53) | 46 (43-50) |
| BMI (kg/m^2^) | 24.9 (22.7-28.2) | 26 (23-29) | 25.5 (23.3-28.1) | 34.3 (30.9-38.7) |
| Disposition index | 763 (456-1,290) | 660 (440-980) | 483 (345-736) | 461 (315-659) |

Raw data are median (interquartile range) or mean±SD. NGT, normal glucose tolerance. IFG, impaired fasting glucose. IGT, impaired glucose tolerance. scT2D, screen detected type 2 diabetes.
