# Supplementary material for: The Type 2 Diabetes Risk Allele of TMEM154-rs6813195 Associates with Decreased Beta Cell Function in a Study of 6,486 Danes
Source: PLoS One. 2015 Mar 23;10(3):e0120890. doi: 10.1371/journal.pone.0120890 (PMC4370672; doi:10.1371/journal.pone.0120890)
Supplement: S5 Table — (DOCX) [file pone.0120890.s005.docx]

**S5 Table**. Measurements of serum insulin and calculation of glycemic indexes

| **Trait** | **Measurement or calculation** |
| --- | --- |
| Serum insulin (excluding des-31,32) | Measured using the AutoDELFIA insulin kit (Perkin-Elmer, Wallac, Turku, Finland) |
| Insulinogenic index | (Serum insulin at 30-min (pmol/l) - fasting serum insulin (pmol/l)) / (plasma glucose at 30-min (mmol/l) - fasting plasma glucose (mmol/l)) |
| ISI_Matsuda_ | (10,000/√(fasting plasma glucose (mmol/l)x18 × fasting serum insulin (pmol/l)/6) × (mean plasma glucose (mmol/l)x18 × mean serum insulin (pmol/l)/6 during OGTT)) |
| Disposition index | The insulinogenic index × ISI_Matsuda_ |
| BIGTT-Si and BIGTT-AIR | The indexes apply information on sex and BMI combined with plasma glucose and serum insulin during OGTT that highly correlate with indexes obtained during an IVGTT and calculated as previously reported [[10](#_ENREF_10)] |
